# ﻿Tadpoles of four sympatric megophryinid frogs (Anura, Megophryidae, Megophryinae) from Mangshan in southern China

**DOI:** 10.3897/zookeys.1139.81641

**Published:** 2023-01-09

**Authors:** Tianyu Qian, Yonghui Li, Jun Chen, Pipeng Li, Daode Yang

**Affiliations:** 1 Institute of Wildlife Conservation, Central South University of Forestry and Technology, Changsha 410004, China Central South University of Forestry and Technology Changsha China; 2 Institute of Herpetology, Shenyang Normal University, Shenyang 110034, China Shenyang Normal University Shenyang China; 3 Administration Bureau of Hunan Mangshan National Nature Reserve, Chenzhou 423000, Hunan, China Administration Bureau of Hunan Mangshan National Nature Reserve Chenzhou China

**Keywords:** Amphibian, integrative taxonomy, larvae, *Megophrys*, Nanling Mountains

## Abstract

Sympatric distribution and potentially long larval development time make the assignment of tadpoles confusing in Asian-horned frogs of the subfamily Megophryinae. In this study, we used molecular data to identify four syntopic megophryinid tadpoles from Mangshan on the border between Hunan and Guangdong provinces in southern China: *Brachytarsophryspopei*, *Boulenophrysshimentaina*, Bo.cf.ombrophila, and *Bo.nanlingensis*. A detailed re-description of the *Br.popei* tadpoles is provided as well as the first descriptions of three *Boulenophrys* tadpoles based on external morphology and coloration. An effort is attempted to distinguish these tadpoles by coloration patterns: the dorsal pattern, ventral pattern, and pattern on tail are useful for field identification of these tadpoles. However, the variation of color pattern could sometimes make species delineation difficult. Researchers are encouraged to document coloration in life with photographs and the collection of tadpoles of different development stages and sizes advocated in order to better understand how color may change during larval development.

## ﻿Introduction

The Asian Horned Frogs in the subfamily Megophryinae Bonaparte, 1850 belong to seven genera and 129 recognized species with wide distributed in Asia, ranging from India and Bhutan to China and south to the Sundas and the Philippines (Frost 2022). Tadpoles of Megophryinae are characterized by a funnel-like oral disc, which allows them to feed beneath the water surface or anchor to a substrate for keeping safe during floods or other threats; this mouthpart specialization had intrigued scientists for almost a century (Hora 1928; Pope 1931; Liu 1950; Huang et al. 1991; Zeng 2021).

Seven monophyletic clades were found within Megophryinae based on molecular analysis (Mahony et al. 2017), previously regarded as seven subgenera of *Megophrys* sensu lato: *Atympanophrys* Tian & Hu, 1983; *Boulenophrys* Fei, Ye and Jiang in Fei & Ye, 2016 (under the name *Panophrys* Rao & Yang, 1997); *Brachytarsophrys* Tian & Hu, 1983; *Megophrys* Kuhl & Van Hasselt, 1822; *Ophryophryne* Boulenger, 1903; *Pelobatrachus* Beddard, 1908; and *Xenophrys* Günther, 1864. Recent taxonomic studies have elevated the seven subgenera to the level of genera (Li et al. 2020; Lyu et al. 2021; Qi et al. 2021). However, the morphological differences remain insufficient for clear delineation of these genera. A recent review of the genus *Brachytarsophrys* by Li et al. (2020) regarded the tadpole ventral pattern as a diagnostic character for species identification. Subsequently, Tapley et al. (2020a) tentatively suggested the presence of “white longitudinal stripe on the ventrolateral surface of the head and body” in tadpoles as a diagnostic character for *Brachytarsophrys*. However, there are no known larval characters that differentiate the remaining megophryinid genera from one another. The external morphology, including oral disc, as well as internal buccal features are highly conserved in all species in this subfamily (Huang et al. 1991; Grosjean 2003). The coloration in life, although rarely described, is considered useful for species identification since Leong and Chou (1998) and has attracted more and more attention (Oberhummer et al. 2014; Poyarkov et al. 2017; Tapley et al. 2017, 2020a, 2020b; Li et al. 2018; Munir et al. 2019; Liu et al. 2020; Shi et al. 2020a, 2020b, 2021; Wu et al. 2020). However, compared to the rate of new megophryinid frog species descriptions, tadpole descriptions remain scarce or are very brief, resulting in insufficient diagnostic characters for comparison and/or field recognition.

Before molecular analysis was widely used in megophryinid taxonomic studies, tadpoles were assigned to species based on their association with post-metamorphic/adult specimens collected from the same site, e.g., *Boulenophrysjingdongensis* (Wang in Wang et al. 2012), *Xenophrysmangshanensis* (Fei & Ye in Fei et al. 1990), tadpoles described by Fei and Ye (2016) and *Xenophryslongipes* (Boulenger, 1886), tadpoles described by Leong and Chou (1998). Notably, many megophryinid species are reported to be in sympatry, including sister species (e.g., Wang et al. 2014, 2019; Chen et al. 2017; Poyarkov et al. 2017; Liu et al. 2018; Mahony et al. 2018, 2020; Shi et al. 2020a; Tapley et al. 2018, 2020b, 2021). Some megophryinid tadpoles are suspected of having long larval development periods of over one year (Grosjean 2003; Tapley et al. 2020a). Consequently, cf. tadpoles collected from the same site with adult frogs may not necessarily belong to the same species. Thus, the description of tadpoles without molecular data has become suspicious if there are any other megophyinid species discovered in sympatry.

Mangshan, a part of Nanling Mountains, is located on the border between Hunan and Guangdong provinces in southern China. The first megophryinid record from this area was a tadpole with a funnel-like oral disc collected at the mountain top of Guangdong/Hunan border (Mell 1922), which was identified as “*Megalophrysboettgeri*” (currently *Boulenophrysboettgeri*). This record has been shown to be erroneous as no adult *B.boettgeri* frogs were found in this area (Shen 1983; Shen et al. 2014), and the horned frogs collected in Mangshan were identified as “*Megophryskuatunensis*” (currently *Bo.kuatunensis*), “*Megophrysbrachykolos*” (currently *Bo.brachykolos*), and *Brachytarsophryscarinense* in “*Fauna Hunan, Amphibia*” (Shen et al. 2014). Subsequently, this “*Brachytarsophryscarinense*” population was described as a new species *Brachytarsophryspopei* Wang, Yang & Zhao in Zhao et al. (2014), while the identity of the two *Boulenophrys* frogs, that were both previously regarded as widespread (e.g., Fei et al. 2009; Fei and Ye 2016) was questioned after molecular analysis based on large-scale sampling (Tapley et al. 2017; Liu et al. 2018; Gao et al. 2022).

In this study, we collected tadpoles bearing funnel-like oral disc from Mangshan. Using DNA barcoding, we confirmed that this collection of syntopic tadpoles was composed of four species: *Brachytarsophryspopei*, *Boulenophrysnanlingensis* (Lyu, Wang, Liu & Wang in Wang et al. 2019), *Bo.shimentaina* (Lyu, Liu & Wang in Lyu et al. 2020), and Bo.cf.ombrophila (Messenger & Dahn in Messenger et al. 2019). Based on the examination of external morphology and coloration in life, we re-described the tadpoles of *Br.popei* and provided the first description of the three *Boulenophrys* tadpoles.

## ﻿Materials and methods

### ﻿Samples

All tadpoles were collected from Mangshan, Yizhang, Hunan Province, China during field surveys in June, July, and November 2021. Specimens were photographed in life in a transparent acrylic box before being euthanized with buffered tricaine methanesulfonate (MS-222) and then fixed with 10% formalin for storage. Tissue samples (tail fin/muscle) were preserved in 95% ethanol for molecular analysis. Specimens were deposited at the Institute of Wildlife Conservation, Central South University of Forestry and Technology (**CSUFT**), Changsha, China.

### ﻿Molecular analysis

Segments of the 16S ribosomal RNA gene (16S) were used for species identification. Primer sequences (L3975 and H4551) from Simon et al. (1994) were used for PCR amplification in 50 µl reaction volumes under the following conditions: 98 °C for 2 min; followed by 30 cycles of 98 °C for 10 sec, 55 °C for 10 sec, and 72 °C for 10 sec, with a final extension step at 72 °C for 5 min. PCR purification and sequencing were performed by Biomarker Technologies Co. (Beijing, China). The new sequences were then searched on BLAST (**NCBI**) to verify their approximate identity. The identification of GenBank accession numbers were retrieved from the BLAST result and manually verified by checking the original references. Uncorrected *p*–distance between the new sequences and sequences from the BLAST result was calculated using MEGA 6 (Tamura et al. 2013). Before calculating uncorrected *p*–distances, sequences were aligned using the MUSCLE algorithm with default parameters (Edgar 2004) and trimmed with gaps partially deleted in MEGA 6.

### ﻿Morphology

Image J 1.53k software (Schneider et al. 2012) was used to measure the tadpoles from photographs of preserved specimens taken next to a scale (10 mm length). The staging follows Gosner (1960), and the terminology for external morphology follows Altig and McDiarmid (1999). Measurements and morphometric abbreviations follow Oberhummer et al. (2014). Definitions of abbreviations are as follows:

**BH** body height, maximal body height at trunk;

**BL** body length from snout to the point where the axis of the tail myotomes meets the body wall;

**BS** body end to the center of spiracle;

**BW** maximal body width;

**ED** eye diameter;

**ES** eye–snout distance;

**IND** the internarial distance measured from center to center;

**IOD** the interorbital distance measured from center to center;

**MTH** maximal tail height;

**LFH** lower fin height at MTH;

**UFH** upper fin height at MTH;

**NE** distance from the center of naris to the center of the eye;

**ODW** oral disc width;

**SN** distance from the center of naris to snout;

**SS** distance from snout to the center of spiracle;

**TTL** total length;

**TAL** tail length = TTL – BL;

**TMH** tail muscle height at the body-tail junction, where ventral line of musculature meets trunk contour;

**TMW** tail muscle width at the same level as TMH.

For the measurement of oral disc width (ODW) in preserved specimens, we expanded the oral disc by anchoring it to a glass (as shown in Fig. 3D).

We compared the tadpoles with their congeneric tadpoles described in the literature where molecular data has been used to confirm species identity: *Br.popei*, tadpoles described by Zhao et al. (2014) and Li et al. (2020); *Br.feae* (Boulenger, 1886), *Br.orientalis* Li, Lyu, Wang & Wang in Li et al. 2020, and *Br.chuannanensis* Fei, Ye & Huang in Fei and Ye 2001, tadpoles described by Li et al. (2020); *Br.intermedia* (Smith, 1921), tadpoles described by Tapley et al. (2020a); *Bo.lini* (Wang & Yang in Wang et al. 2014); *Bo.fansipanensis* (Tapley, Cutajar, Mahony, Nguyen, Dau, Luong, Le, Nguyen, Nguyen, Portway, Luong & Rowley, 2018), *Bo.hoanglienensis* (Tapley, Cutajar, Mahony, Nguyen, Dau, Luong, Le, Nguyen, Nguyen, Portway, Luong & Rowley, 2018), and *Bo.jingdongensis* (Fei & Ye in Fei et al. 1983), tadpoles described by Tapley et al (2020b); *Bo.baishanzuensis* (Wu, Li, Liu, Wang & Wu, 2020); *Bo.lushuiensis* (Shi, Li, Zhu, Jiang, Jiang & Wang, 2021); *Bo.rubrimera* (Tapley, Cutajar, Mahony, Chung, Dau, Nguyen, Luong & Rowley, 2017); *Bo.jiangi* (Liu, Li, Wei, Xu, Cheng, Wang & Wu, 2020); and *Bo.leishanensis* (Li, Xu, Liu, Jiang, Wei & Wang, 2018). We further summarized the morphological characteristics of all megophryinid tadpoles that were identified based on molecular data as described in the literature: *Atympanophrysgigantica* (Liu, Hu & Yang, 1960), tadpoles described by Tapley et al. (2020b); *Ophryophryneelfina* Poyarkov, Duong, Orlov, Gogoleva, Vassilieva, Nguyen, Nguyen, Nguyen, Che & Mahony, 2017; *Pelobatrachuskalimantanensis* (Munir, Hamidy, Matsui, Iskandar, Sidik & Shimada, 2019); *Xenophrysmedogensis* (Fei, Ye & Huang, 1983), X.cf.pachyproctus (Huang in Huang and Fei 1981), and *X.yeae* (Shi, Zhang, Xie, Jiang, Liu, Ding, Luan & Wang, 2020), tadpoles described by Shi et al. (2020a); *X.maosonensis* (Bourret, 1937), tadpoles described by Tapley et al. (2020b); *X.serchhipii* Mathew & Sen, 2007, tadpoles described by Raj et al. (2022); *X.lekaguli* (Stuart, Chuaynkern, Chan-ard & Inger, 2006); “*X.katabhako*” Deuti, Grosjean, Nicolas, Vasudevan & Ohler, 2017 (synonymized to *X.monticola* Günther, 1864 by Mahony et al. 2018); *X.periosa* (Mahony, Kamei, Teeling & Biju, 2018), tadpoles described by Shi et al. (2020b); and “*Megophrys*” *dringi* Inger, Stuebing & Tan, 1995, tadpoles described by Oberhummer et al. (2014).

## ﻿Results

### ﻿Tadpole identification

Two tadpoles were identified as *Brachytarsophryspopei* based on an uncorrected *p*–distance of 0.0–0.7% from the samples in the type series from Hunan, Guangdong, and Jiangxi Provinces (GenBank accession numbers: KM504251–KM504258). Three tadpoles from the same collection site and bearing the same characteristics as the above two tadpoles but without molecular data were also assigned to *Br.popei*. The collection site of these tadpoles is only ~ 5 km from the collection site of paratype SYS a00589 (GenBank accession number: KM504051) in the adjacent Nanling Nature Reserve, Guangdong Province.

A total of 14 tadpoles was identified as *Boulenophrysnanlingensis*, which exhibited an uncorrected *p*–distance of 0.0–0.4% from the holotype SYS a001964 (GenBank accession number: MH406646) collected ~ 10 km in the adjacent Nanling Nature Reserve, Guangdong Province.

Five tadpoles were identified as *Boulenophrysshimentaina*, which exhibited an uncorrected *p*–distance of 1.2% from the type series (GenBank accession numbers: MH406655–MH406656, and 787–788) collected from Shimentai Nature Reserve, Guangdong Province, China ~ 70 km to the south of Mangshan. This genetic distance is smaller than the interspecies *p*–distance in the subfamily Megophryinae used to identify tadpoles (1.4% in *Br.intermedia*, Tapley et al. 2020a).

Four tadpoles showed an uncorrected *p*–distance of 1.8–2.1% from the type series of *Bo.ombrophila* (GenBank accession numbers: KX856397–KX856404) collected from Wuyishan, Fujian Province, China ~ 500 km to the northeast of Mangshan. This genetic distance almost equals to the threshold (2.0% in 16S gene, proposed by Chen et al. 2017) for a potential new species in this subfamily. However, the other two populations of *Bo.ombrophila* suspected by Messenger et al. (2019) from Jiulianshan, Jiangxi Province (*Megophrys* sp 8 in Liu et al. 2018) and Renhua County, Guangdong Province (*M.* sp 9 in Liu et al. 2018) were closer to our samples both in the geographical distance of collection sites and genetic distances based on 16S gene. The Jiulianshan population of *Bo.ombrophila* (GenBank accession numbers: MH406836–MH406840) collected from ~ 160 km to the east of Mangshan showed an uncorrected *p*–distance of 1.6–1.8% from our samples. The Renhua population of *Bo.ombrophila* (GenBank accession numbers: MH406650–MH406653, and MH406834) collected from ~ 70 km to the east of Mangshan showed an uncorrected *p*–distance of 1.4–1.6% from our samples. Further examination of adult frogs revealed morphometric differences between the Mangshan population and the type series from Wuyishan, Fujian. However, the morphometric and morphological data were unavailable for the Jiulianshan population and Renhua Population. Thus, we currently identified the Mangshan population as Bo.cf.ombrophila.

### ﻿Morphological description

All examined specimens exhibited a funnel-like oral disc corresponding to the tadpole description of “*Megophrysminor*” by Liu (1950): “The mouth is terminal, with a large funnel that has two long lateral wings, a short ventral wing and a comparatively narrow convex flap above. The tips of the lateral and ventral wings are bluntly pointed.” Detailed tadpole descriptions are given below.

#### ﻿ Brachytarsophrys popei

Taxon classificationAnimaliaAnuraMegophryidae

8B3CEC9D-95F1-5A1E-82CC-E55065A68026

Fig. 1

##### Remark.

The following description is based on five tadpoles at Stages 26–27 (*N* = 2) and 36–37 (*N* = 3). Body ratio ranges represent all specimens. Raw measurements are given in Table 1.

**Table 1.** Morphometric data of the tadpole specimens used in this study. For abbreviations, see Materials and Methods. “*” indicates specimens with broken tails, and “\” indicates “no data”.

Species	Voucher No.	Stage	TTL	BL	TAL	BH	SS	BS	ED	TMH	MTH	UFH	LFH	IOD	IND	NE	SN	ES	TMW	BW	ODW
*Brachytarsophryspopei*	CSUFT T10944	27	31.8	9.0	22.8	4.2	5.0	4.2	1.2	3.0	5.4	1.7	1.9	3.8	2.5	1.1	1.6	2.7	2.7	4.8	5.4
	CSUFT T10945	26	27.9	7.9	20.0	3.9	4.9	3.4	1.2	2.9	5.2	1.4	1.6	3.8	2.6	1.1	1.5	2.6	2.5	4.4	5.9
	CSUFT T10115	37	35.0	10.5	24.5	4.7	6.1	4.5	1.4	3.1	6.4	2.1	2.0	4.6	2.8	1.4	1.9	3.3	2.9	5.8	7.4
	CSUFT T10117	37	37.3	11.0	26.3	5.0	6.3	4.7	1.5	2.9	5.8	1.9	1.9	4.7	2.8	1.5	2.0	3.4	2.9	5.9	7.8
	CSUFT T10119	36	36.9	10.9	26.0	5.0	6.3	4.9	1.4	3.2	6.3	2.1	2.1	4.7	3.0	1.5	1.8	3.2	3.1	5.9	7.6
*Boulenophrysshimentaina*	CSUFT T10156	25	28.5	7.3	21.2	3.1	3.9	3.8	0.8	2.3	4.4	1.2	1.1	3.1	2.2	1.0	1.4	2.3	2.4	4.0	5.2
	CSUFT T10277	26	28.6	8.0	20.6	3.8	4.6	3.5	1.0	2.5	4.5	1.3	1.3	3.4	2.4	1.0	1.6	2.6	2.4	4.4	6.3
	CSUFT T10279*	25	\	8.3	\	3.7	4.7	3.9	1.0	2.6	\	\	\	3.4	2.3	1.0	1.5	2.4	2.5	4.3	6.6
	CSUFT T10285	27	28.5	8.1	20.4	3.7	4.7	3.6	1.0	2.8	5.6	1.5	1.5	3.4	2.3	1.0	1.5	2.4	2.4	4.4	5.7
	CSUFT T10283	28	27.0	8.0	19.0	3.5	4.4	3.7	0.9	2.6	4.8	1.3	1.2	3.2	2.3	0.9	1.4	2.3	2.2	4.1	6.0
*Boulenophrysnanlingensis*	CSUFT T10144	25	18.7	5.4	13.3	2.3	3.2	2.5	0.7	1.7	2.7	0.7	0.8	2.3	1.6	0.7	1.0	1.7	1.5	2.8	3.8
	CSUFT T10986	35	40.1	10.8	29.3	5.4	6.4	4.5	1.6	3.7	7.3	1.7	1.6	4.7	3.2	1.5	2.4	3.7	3.6	5.7	7.8
	CSUFT T10969	34	34.4	8.8	25.6	4.0	5.6	4.2	1.3	3.0	5.5	1.4	1.3	3.9	2.7	1.1	1.9	3.0	2.8	4.8	5.6
	CSUFT T10261	25	25.1	6.7	18.4	3.1	4.2	2.7	0.8	1.8	4.0	1.2	1.2	2.7	1.9	0.8	1.2	2.0	1.5	3.8	5.4
	CSUFT T10262	25	27.2	6.5	20.7	2.8	4.0	2.8	1.0	1.9	4.2	1.2	1.2	2.9	1.9	0.9	1.2	2.1	1.7	3.6	5.2
	CSUFT T10273	28	35.7	9.4	26.3	4.4	5.4	4.3	1.1	3.2	5.9	1.5	1.5	4.0	2.8	1.0	1.8	2.9	3.0	5.0	7.9
	CSUFT T10991	27	39.1	10.3	28.8	4.4	6.0	4.4	1.3	3.1	6.7	2.0	1.8	4.0	2.8	1.2	1.9	3.1	2.8	5.3	7.6
	CSUFT T10284	25	18.9	5.3	13.6	2.6	3.3	2.2	0.8	1.7	3.4	1.0	0.9	2.6	1.6	0.8	1.0	1.8	1.6	3.2	4.1
	CSUFT T10302*	25	\	7.3	\	3.3	4.0	3.3	0.9	2.4	\	\	\	3.0	2.0	0.9	1.3	2.2	2.1	4.0.	6.0
	CSUFT T10303	25	26.2	6.8	19.4	3.3	3.9	3.1	0.8	2.3	4.0	1.3	1.2	2.9	1.9	0.9	1.3	2.2	2.0	3.9	4.9
	CSUFT T10377	27	28.1	8.2	19.9	3.5	4.9	3.4	1.0	2.3	5.3	1.5	1.5	3.4	2.3	0.9	1.6	2.4	2.0	4.4	6.8
	CSUFT T10378	28	26.9	8.2	18.7	3.4	5.0	3.2	1.2	2.4	5.2	1.3	1.4	3.4	2.2	0.9	1.4	2.3	2.2	4.2	5.9
	CSUFT T10376	27	24.8	7.0	17.8	3.5	4.1	3.1	1.0	2.1	4.8	1.4	1.4	3.1	2.1	0.9	1.3	2.1	2.0	4.0	5.7
	CSUFT T10379	29	27.8	7.7	20.1	3.5	4.5	3.1	1.2	2.5	5.0	1.3	1.4	3.1	2.2	1.0	1.4	2.3	1.9	4.0	5.7
Boulenophryscf.ombrophila	CSUFT T10270	36	33.7	10.0	23.7	4.5	5.6	4.6	1.4	2.9	6.0	1.4	1.6	4.2	2.8	1.4	1.7	3.0	2.9	5.1	8.3
	CSUFT T10272	27	33.1	8.9	24.2	4.1	5.3	4.2	1.2	3.0	6.0	1.4	1.4	3.8	2.5	1.1	1.7	2.8	2.8	4.8	7.8
	CSUFT T10288	26	30.4	8.4	22.0	3.9	4.8	3.8	1.1	2.7	5.9	1.6	1.4	3.6	2.4	1.1	1.5	2.6	2.6	4.6	7.2
	CSUFT T10992	25	20.9	5.1	15.8	2.1	2.8	2.0	0.5	1.6	3.0	0.8	0.8	2.2	1.5	0.6	1.0	1.7	1.3	2.7	3.9

##### Specimens examined.

CSUFT T10115 (Stage 37, Field voucher: MT05; GenBank accession number: ON209276), CSUFT T10117 (Stage 37; Field voucher: MT07; GenBank accession number: ON209284), and CSUFT T10119 (Stage 36; Field voucher: MT09; not sequenced), collected on 30 May 2021 from Tiantaishan (24.972277°N, 112.963394°E, ca. 1280 m a.s.l.), Mangshan, Hunan Province, China; and CSUFT T10944 (Stage 27, Field voucher: MT1104; not sequenced), and CSUFT T10945 (Stage 26; Field voucher: MT1105; not sequenced), collected on 16 November 2021 from the same site as the first specimens.

##### External morphology.

The body is oval, robust, and flattened above (BW/BL 53.3–55.7% at Stages 26–27, *N* = 2; and 53.6–55.2% at Stages 36–37, *N* = 3); the head is wider than the trunk; the eyes are located dorsolaterally, the pupils are round; the nares are oval, opening laterally, closer to the eye than to the tip of the snout (NE/SN 68.8–73.3% at Stages 26–27, *N* = 2; and 73.7–83.3% at Stages 36–37, *N* = 3); the internarial distance is smaller than the interorbital distance (IND/IOD 65.8–68.4% at Stages 26–27, *N* = 2; and 59.6–63.8% at Stages 36–37, *N* = 3); the rims of nares are raised from the body wall and directed posterolaterally; the spiracle is sinistral and low on the left flank; the spiracle tube is short, protruding posterodorsally, free from the body at the tip, and opening posterolaterally (SS/BL 55.6–62.0% at Stages 26–27, *N* = 2; and 57.3–58.1% at Stages 36–37, *N* = 3); the anal tube opens medially, unattached to the ventral fin; the dorsal fin arises behind the body-tail junction while the ventral fin is connected to the trunk; the tail muscle is massive, taller than tail fins before reaching the maximum tail height (TMH/MTH 55.6–55.8% at Stages 26–27, *N* = 2; and 48.4–50.8% at Stages 36–37, *N* = 3), and the tail tip is bluntly pointed, the tail length accounts for 71.7% (at Stages 26–27, *N* = 2) and 70.5–70.5% (at Stages 36–37, *N* = 3) of the total length; the mouth is terminal and the oral disc is funnel-like (BW/ODW 74.6–88.9% at Stages 26–27, *N* = 2; and 75.6–78.4% at Stages 36–37, *N* = 3); three and four rows of short oval submarginal papillae can be observed on the upper lip and lower lip, respectively; keratodonts are absent; the upper jaw sheath is brush-like, exhibiting a small median notch, while the lower jaw sheath is thin, sickle-shaped, weakly keratinized, and finely serrated.

##### Coloration.

In life, the background color of the head and trunk is dark brown; the dorsal pattern is pale brown interspersed with dark brown chromocytes, extending to above the horizontal level of the spiracle on the trunk from a lateral perspective; the dorsal surface of the anterior part of the tail is pale brown marbled with dark brown speckles; neuromasts are distinctly visible on the head, trunk and tail; the region between the anterior edges of the eyes and the median point of the upper lip is pigmented with a dark brown V-shaped pattern; the narial rims are pale brown; the oral disc is golden-pigmented, with a translucent edge; the submarginal papillae on lips are dark brown-pigmented. Laterally, the tail is pale brown-pigmented; dense goldish spots are located at the anterior part of the lateral surface of tail muscle, becoming smaller and at the middle, then disappearing posteriorly; three distinct dark brown stripes extended from the body-tail junction, and horizontally positioned at the anterior part of the tail; the upper and lower stripes end before reaching the maximum tail height, while the middle stripe is about half the length of the others; the upper and middle stripes are incomplete; the anterior part of the upper fin is opaque, marbled with goldish pigmentation and brown speckles; the anterior part of the ventral fin, as well as the anal tube are semi-translucent with dense large golden spots; the rest of the fins are semi-translucent, and exhibit sparse dark brown speckles interspersed with small goldish dots. The ventral surface of the body is rather dark; the belly is dark purplish covered with dense white spots; two longitudinal stripes, positioned ventrolaterally, extending from the snout to the vertical edge of the eyes posteriorly, and sometimes appear to broken; a transverse bar is positioned at the head-trunk junction of the vertical edge of the anterior spiracle and is always interrupted at the middle; the spiracle region and the corresponding region on the other side of the body, are covered with a short white stripe, that starts from the head-body connection, and terminated before reaching the region of the spiracle tube opening; regions without white pigmentation have less melanocytes; the gills and gut coils are indistinctly visible through the ventral skin. The eye sclera is silver with black dots; the iris periphery is wide and black; the iris is golden sprinkled with black dots; and the spiracle is translucent without pigmentation. In tadpoles at Stages 36–37, the hindlimbs are semi-transparent, and the outer aspect of the legs exhibits brown pigmentation interspersed with goldish chromocytes.

In preserved specimens, the tail stripes are still prominent; an incomplete V-shaped pigmentation pattern is still visible; the ventral pattern is translucent milky white; the golden pigmentation wanes on the oral disc; and the hindlimb bones are visible in ventral view in Stage 36–37 tadpoles.

**Figure 1.**
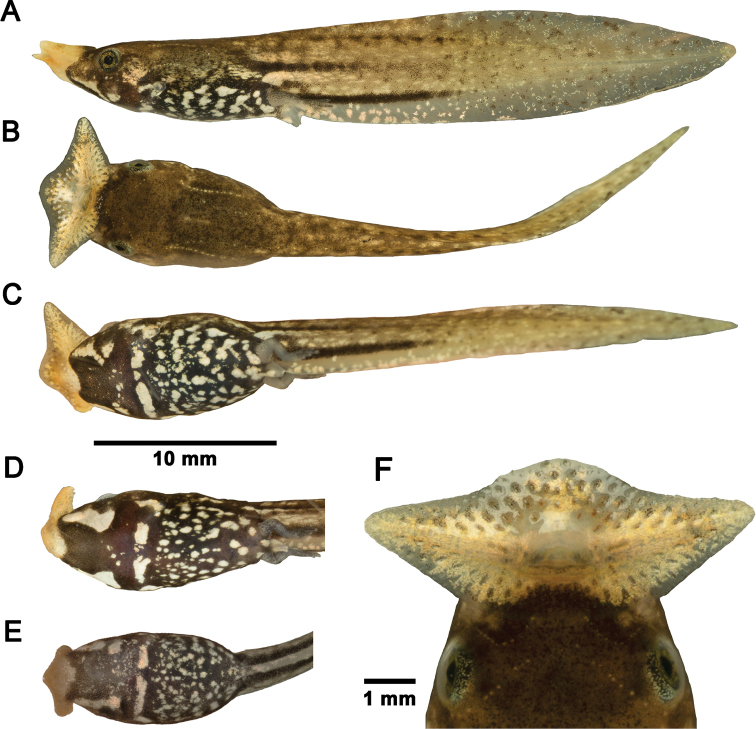
*Brachytarsophryspopei* tadpoles in life **A–C** tadpole CSUFT T10117 (Stage 37) lateral view, dorsal view, and ventral view **D** ventral pattern of tadpole CSUFT T10119 (Stage 36) **E** ventral pattern of tadpole CSUFT T10945 (Stage 26); and **F** oral disc of tadpole CSUFT T10117 (Stage 37). **D** and **E** share the same scale bar with **A–C**.

##### Comparisons.

Tadpoles of *Br.popei* differ significantly from the three syntopic *Boulenophrys* tadpoles described below by the unique pattern of two longitudinal white ventrolateal stripes on head, a transverse white bar on chest, and distinct large spots on belly (vs. absence of stripes and bars, and smaller spots/speckles on belly).

The differences in ventral pattern between four *Brachytarsophrys* tadpoles were compared by Li et al. (2020) and summarized in Table 2. The tadpole of *Br.popei* (Stage 29, *N* = 1) illustrated in their paper (also in Zhao et al. 2014, but marked as Stage 27), which was collected ~ 200 km north of Mangshan has a complete transverse white ventral bar. In contrast, our tadpoles (Stages 26–27, *N* = 2; and Stages 36–37, *N* = 3) consistently exhibit an interrupted white transverse ventral bar. This difference may be due to geographic variation or insufficient sample size. However, the presence of a transverse bar on chest could distinguish *Br.popei* tadpoles from *Br.orientalis* and *Br.intermedia* (vs. absent in both). In addition, the width of the transverse bar is markedly smaller than that in *Br.chuannanensis* (see Li et al. 2020: fig. 5E, F). Furthermore, compared with *Br.intermedia*, the tadpoles of *Br.popei* have a distinctly smaller size at Stage 36 (TTL 36.9 mm vs. 48.7 mm). Zhao et al. (2014) illustrated a metamorph of *Br.feae* at Stage 44 with several short stripes on belly (vs. spots or speckles in *Br.popei*, *Br.orientalis*, and *Br.chuannanensis*). We believe this pattern should be confirmed using more specimens at an earlier developmental stage in case this is a transitional form during metamorphosis. Further comparisons between *Br.popei* tadpoles and all megophryinid tadpoles that were identified using molecular data are shown in Tables 2 and 3.

**Table 2.** Comparison of color pattern among tadpoles of the subfamily Megophryinae which were identified based on molecular data. “*” indicates characteristics not mentioned in the text but were illustrated in the figure, and “\” indicates “no data”.

Species	Stage	Neuromasts visibility	Intestine visibility	Dorsum pattern	Pattern on tail	Ventral pattern	References
***Atympanophrys***							
*A.gigantica*	35, *N* = 5	visible	visible	uniform dark brown	pale yellowish brown without speckles	translucent dark grey and speckled with white	Tapley et al. 2020b
***Brachytarsophrys***							
*Br.popei*	26–27, and 36–37, *N* = 5	distinct	indistinct	uniform dark brown	small dots and longtitudinal stripes	ventrolateral stripes on head and body, incomplete transverse bar on chest, dense large spots on belly	This study
	26–29, *N* = 14	\	\	\	three dark longitudinal stripes	two longitudinal white stripes along the sides of body, a completed transverse bar on chest, belly mottled with dense white speckles	Zhao et al. 2014
*Br.intermedia*	32, 36, and 39, *N* = 4	pale brown	not visible	pale brown with a darker brown medial saddle	speckled with dark brown, and longitudinal stripes	ventrolateral stripes on head and body, small spots on chest and belly	Tapley et al. 2020a
*Br.chuannanensis*	38, *N* = 1	\	\	\	distinct dark longtitudinal stripes*	wide ventrolateral stripes on head*; wide transverse bar on chest; and several spots on belly*	Li et al. 2020
*Br.orientalis*	36, *N* = 1	\	\	brown	three short dark longitudinal stripes	two short, longitudinal white stripes on sides of ventral surface of head and body; absence of transversal white stripe on chest; belly mottled with dense white speckles	Li et al. 2020
*Br.feae*	44, *N* = 1	\	\	\	\	transeverse bar on chest; several several transeverse stripes on belly	Li et al. 2020
***Boulenophrys***							
*Bo.shimentaina*	25–28, *N* = 5	distinct	visible	brown with dark brown reticulation	pigmented with dense dark brown markings posteriorly	milky white ventrolateral spots on chest, dense indistinct small milky white speckles on belly	This study
Bo.cf.ombrophila	25, *N* = 1 (TTL 20.9 mm)	indistinct	distinct	pale brown, scattered with dense dark melanocytes	pigmented orange and dark brown speckles	belly covered with dense melanocytes	This study
	26–27, and 36, *N* = 3 (TTL 30.4–33.1 mm)	distinct	indistinct	brown pattern along mid-vertical line	several large brown spots along tail muscle	gold-pigmented white ventrolateral spots on chest, dense white speckles on belly	This study
*Bo.nanlingensis*	25, *N* = 2 (TTL 18.7–18.9 mm)	distinct	distinct	yellowish with pale orangish blotches, or brown with whitish patterns	many brown speckles	gold-pigmented white ventrolateral spots on chest, sparse white speckles on belly	This study
	25, *N* = 3 (TTL 25.1–27.2 mm)	distinct	distinct	pale brown with dark brown pigmentation	many brown speckles	gold-pigmented white ventrolateral spots on chest, sparse white speckles on belly	This study
	27–29, *N* = 4, TTL 24.8–28.1 mm)	distinct	distinct	bi-colored dorsum of pale brown anteriorly and dark brown posteriorly	many brown speckles	gold-pigmented white ventrolateral spots on chest, sparse white speckles on belly	This study
	27–28, and 34–35, *N* = 4, (TTL 35.7–44.4 mm)	distinct	distinct	uniform brownish	many brown speckles	gold-pigmented white ventrolateral spots on chest, sparse white speckles or dense large spots on belly	This study
*Bo.fansipanensis*	25, *N* = 2	obvious	visible	brown with dark brown speckles	small spots and dark brown speckles	a translucent grey brown and speckled with metallic blue and flecked with dark brown	This study
*Bo.jingdongensis*	25, *N* = 1	indistinct	visible	dark brown with cream blotches, bordered by orange flecks	many dark brown speckles	grey brown and speckled with metallic blue	Tapley et al. 2020b
*Bo.hoanglienensis*	26, *N* = 1	distinct	visible	dark brown with reddish brown blotches and reticulated blackish brown	many dark brown speckles	speckled with metallic grey blue flecks	Tapley et al. 2020b
*Bo.rubrimera*	37, *N* = 1	obvious	\	brown with darker speckles	pale yellowish brown with speckles	speckled white and brown	Tapley et al. 2017
*Bo.baishanzuensis*	31, *N* = 1	\	\	brownish black	small white and black spots	\	Wu et al. 2020
*Bo.lushuiensis*	26–27, 32, and 36, *N* = 5	\	visible	brown without distinct patterns	pale brown with dozens of small dark brown patches	scattered with silver tiny patches	Shi et al. 2021
*Bo.leishanensis*	25–26, *N* = 6	visible*	\	yellow-brown	pale colored on fins, and small black spots on tail muscle	dense small white speckles*	Li et al. 2018
*Bo.jiangi*	26, *N* = 2	\	\	yellow-brown	few dark spots on posterior tail muscle*	\	Liu et al. 2020
***Ophryophryne***							
*O.elfina*	25, *N* = 5	visible	not visible	uniform brownish red or brownish orange	few round blackish spots on tail	pale brownish orange, intestine	Poyarkov et al. 2017
***Pelobatrachus***							
*P.kalimantanensis*	30, and 36, *N* = 2	visible*	not visible*	conspicuous dark brown and gold or orange brown pigmentation	marbled with dark brown pigmentation, edges of fins with golden iridophores	belly milky-white pigmented, pale stripe below spiracle extends laterally to half of abdomen*	Munir et al. 2019
	45, *N* = 1	invisible*	not visible*	dark brown without orange gold pigmentation	dark brown	dark brown marbled pattern	Munir et al. 2019
***Xenophrys***							
*X.medogensis* (low-elevation)	35, and 38, *N* = 2	\	\	pale yellow-brown	mottled with pale colored patches	without white patches	Shi et al. 2020a
*X.medogensis* (high-elevation)	27, *N* = 1	\	\	deep brown with copper pigmentation	brown, scattered with tiny white pigment spots, no dark brown patches on tail	semitransparent brown, covered with small white pigments	Shi et al. 2020a
X.cf.pachyproctus	25, *N* = 1	\	\	yellow-brown with two golden spots on dorsalateral mid body	\	\	Shi et al. 2020a
*X.yeae*	28–29, and 31–35, *N* = 9	\	\	brown with dense copper pigments	above lower fin mottled with copper patches	semi-transparent	Shi et al. 2020a
*X.maosonensis*	25, *N* = 2	obvious	visible	brown with dark brown speckles posteriorly	few dark brown speckles	speckled with metallic grey blue	Tapley et al. 2020b
*X.lekaguli*	25, 37–38, and 42, *N* = 6	\	\	pale gray (in preservative)	proximal half of caudal muscle with two or three irregular dark streaks, fins distinctly pigmented only in distal portions (in preservative)	small black spots (in preservative)	Stuart et al. 2006
*X.serchhipii*	32, 34, and 36–38, *N* = 11	\	visible	dark brown (in preservative)	translucent and grey (in preservative)	dark brown, fins are opaque and speckled (in preservative)	Raj et al. 2022
*X.monticola*	25, *N* = 5	\	\	grey olive-green with irregular melanophores (in preservative)	densely arranged melanophores (in preservative)	immaculate, slightly translucent with some rare spots of melanophores (in preservative)	Deuti et al. 2017
*X.periosa*	27, *N* = 1	\	\	greyish brown	dense small speckles	translucent greyish brown	Shi et al. 2020b
	34, *N* = 1	\	\	greyish brown	large spots alongside anterior 2/3 of tail muscle	translucent greyish brown	Shi et al. 2020b
**Incertae sedis with Megophryinae**							
“*Megophrys*” *dringi*	25, *N* = 4	\	visible	conspicuous pattern of intense dark brown and gold pigmentation	pigmented dark brown, interspersed with pale golden iridophores	milky translucent with a few irregularly shaped golden spots	Oberhummer et al. 2014

**Table 3.** Comparison of morphological characteristics among tadpoles of the subfamily Megophryinae, which was identified based on molecular data. “*” indicates characteristics not mentioned in the text but were illustrated in the figure, and “\” indicates “no data”.

Species	Stage	*N*	TTL	BL	TAL/TTL	BW/ODW (expanded)	Mouthpart shape	Narial rim	Tail tip	References
***Atympanophrys***										
*A.gigantica*	35	5	50.7 (42.6–54.9)	16.9 (15.7–18.0)	66.6 (63.2–68.4)	62.6, *N* = 1	hastate	serrated and raised	broadly rounded	Tapley et al. 2020b
***Brachytarsophrys***										
*Br.popei*	26–27	2	36.4±1.2 (35.0–37.3)	8.5±0.8 (7.9–9.0)	71.7(–)	81.7±10.1 (74.6–88.9)	bi-triangular	raised	bluntly pointed	This study
	36–37	3	36.4±1.2 (35.0–37.3)	10.8±0.3 (10.5–11.0)	70.3±0.3 (70.0–70.5)	77.2±1.4 (75.6–78.4)	bi-triangular	raised	bluntly pointed	This study
	26–27	12	\	\	\	\	\	\	bluntly pointed	Zhao et al. 2014
	29	2	\	\	\	\	\	\	bluntly pointed	Zhao et al. 2014
*Br.intermedia*	32	2	45.0±4.7 (41.7–48.3)	14.0±2.2 (12.4–15.5)	69.7±1.7 (67.9–70.3)	\	bi-triangular	raised	pointed	Tapley et al. 2020a
	36	1	48.7	15.0	69.2	50.6	bi-triangular	raised	pointed	Tapley et al. 2020a
	39	1	55.1	16.3	70.4	\	bi-triangular	raised	pointed	Tapley et al. 2020a
*Br.orientalis*	36	1	33.9	12.3	69.2	\	\	\	pointed	Li et al. 2020
***Boulenophrys***										
*Bo.fansipanensis*	25	2	30.8 (26.5–35.0)	9.1 (7.4–10.8)	69.1–72.1	64.8, *N* = 1	bi-triangular	serrated and raised	pointed	Tapley et al. 2020b
*Bo.jingdongensis*	25	1	27.9	8.9	68.1	80.4, *N* = 1	bi-triangular	serrated and raised	rounded	Tapley et al. 2020b
*Bo.hoanglienensis*	26	1	26.5	7.1	73.2	79.3, *N* = 1	bi-triangular	serrated and raised	pointed	Tapley et al. 2020b
*Bo.shimentaina*	25–27	4	28.5±0.1 (28.5–28.6)	7.9±0.4 (7.3–8.3)	72.7±1.5 (71.6–74.4)	72.3±5.8 (65.2–77.2)	bi-triangular	serrated and raised	bluntly pointed	This study
	28	1	27	8	70.4	68.3	bi-triangular	serrated and raised	bluntly pointed	This study
Bo.cf.ombrophila	25	1	20.9	5.1	75.6	69.2	bi-triangular	serrated and raised	bluntly rounded	This study
	26–27	2	31.8±1.9 (30.4–33.1)	8.7±0.4 (8.4–8.9)	72.7±0.5 (72.4–73.1)	62.7±1.7 (61.5–63.9)	bi-triangular	serrated and raised	sharply pointed	This study
	36	1	33.7	10.0	70.3	61.4	bi-triangular	serrated and raised	sharply pointed	This study
*Bo.nanlingensis*	25–27	9	26.0±6.4 (18.7–39.1)	7.1±1.5 (5.3–10.3)	72.8±1.8 (70.8–76.1)	71.4±4.9 (64.7–79.6)	bi-triangular	serrated and raised	pointed	This study
	28–29	3	30.1±4.8 (26.9–35.7)	8.4±0.9 (7.7–9.4)	71.8±2.1 (69.5–73.7)	68.2±4.2 (63.3–71.2)	bi-triangular	serrated and raised	pointed	This study
	34	1	34.4	8.8	74.4	85.7	bi-triangular	serrated and raised	pointed	This study
	35	1	40.1	10.8	73.1	73.1	bi-triangular	serrated and raised	pointed	This study
*Bo.lushuiensis*	26–27	3	27.8±4.0 (23.1–30.3)	8.0±1.1 (6.8–8.8)	70.2±1.9 (68.0–71.3)	66.8±11.0 (56.1–78.0)	\	\	\	Shi et al. 2021
	32	1	42.7	12.1	71.9	57.9	bi-triangular*	\	rounded*	Shi et al. 2021
	36	1	41.1	11.3	72.5	58.4	\	\	\	Shi et al. 2021
*Bo.rubrimera*	37	1	33.3	10.5	68.5	\	\	\	rounded	Tapley et al. 2017
*Bo.baishanzuensis*	31	1	22.7	\	64.8	\	bi-triangular*	\	pointed	Wu et al. 2020
*Bo.leishanensis*	25–27	6	29.7±2.3 (27.0–33.0)	\	64.2±2.1 (61.5–66.7)	\	\	\	pointed	Li et al. 2018
*Bo.jiangi*	26	2	25.5–26.0	\	65.5–70.4	\	bi-triangular*	\	pointed	Liu et al. 2020
*Bo.lini*	28	not provided	\	\	\	\	\	raised	pointed	Wang et al. 2014
	31–34	not provided	\	\	\	\	\	raised	pointed	Wang et al. 2014
***Ophryophryne***										
*O.elfina*	25	5	28.4±1.3 (27.4–30.2)	8.6±0.1 (8.4–8.7)	\	\	bi-triangular*	“nares tubular”	bluntly rounded	Poyarkov et al. 2017
***Pelobatrachus***										
*P.kalimantanensis*	30	1	38.9	11.2	71.2	\	\	\	blunt	Munir et al. 2019
	36	1	47.0	12.9	72.6	\	\	\	blunt	Munir et al. 2019
	45	1	31.2	13.5	56.7	\	\	\	\	Munir et al. 2019
***Xenophrys***										
*X.yeae*	28–29	4	34.3±0.4 (33.9–34.8)	10.6±0.3 (10.2–11.0)	69.0±1.2 (67.3–69.9)	70.6±6.1 (64.8–78.0)	\	\	\	Shi et al. 2020a
	31–34	4	34.9±1.1 (33.7–35.8)	11.0±0.5 (10.4–11.4)	68.4±0.5 (68.0–69.1)	78.6±13.7 (66.2–92.9)	\	\	\	Shi et al. 2020a
	35	1	38.4	10.9	71.6	66.2	\	\	rounded*	Shi et al. 2020a
X.cf.pachyproctus	25	1	19.1	6.1	68.1	63.3	\	\	bluntly pointed*	Shi et al. 2020a
*X.medogensis* (high-elevation)	27	1	33.7	9.5	71.5	98.1	\	\	pointed*	Shi et al. 2020a
*X.medogensis* (low-middle elevation)	35	1	42.7	13.3	68.9	85.2	\	\	pointed*	Shi et al. 2020a
	38	1	43.6	13.2	69.5	83.1	\	\	\	Shi et al. 2020a
*X.maosonensis*	25	2	35.5 (34.4–36.6)	8.8 (8.1–9.5)	76.5–77.9	73.2	bi-triangular	raised	narrowly rounded	Tapley et al. 2020b
*X.lekaguli*	25	2	\	9.0–10.4	\	\	\	not raised	rounded	Stuart et al. 2006
	37	2	\	12.1–12.9	\	\	\	not raised	rounded	Stuart et al. 2006
	38	1	\	13.8	\	\	\	not raised	rounded	Stuart et al. 2006
	42	1	\	14.2	\	\	\	not raised	rounded	Stuart et al. 2006
*X.serchhipii*	32	1	28.6	10	65.0	\	\	\	\	Raj et al. 2022
	34	4	29.9±1.40	10.2±0.30	\	\	\	\	\	Raj et al. 2022
	36	4	29.3±0.47	11.3±0.11	72, *N* = 1	\	\	“an elevated projection”	pointed	Raj et al. 2022
	37	1	28.9	11.9	58.8	\	\	\	\	Raj et al. 2022
	38	1	35.6	13.0	63.5	\	\	\	\	Raj et al. 2022
*X.monticola*	25	7	24.7±2.7 (21.1–28.1), *N* = 5	6.9±0.9 (5.9–8.2)	70–71, *N* = 4	\	\	“waves”	finely rounded	Deuti et al. 2017
*X.periosa*	27	3	30.4±1.5 (29.0–32.0)	8.9±0.1 (8.4–9.5)	70.7±0.4 (70.3–71.0)	60.3±3.6 (58.2–64.5)	bi-triangular*	\	bluntly pointed	Shi et al. 2020b
	34	3	47.3±4.4 (42.7–51.4)	12.8±0.9 (12.1–13.8)	72.9±1.1 (71.7–73.9)	75.8±5.9 (69.9–81.6)	bi-triangular*	\	bluntly pointed	Shi et al. 2020b
**Incertae sedis with Megophryinae**										
“*Megophrys*” *dringi*	25	4	32.28±6.05 (23.23–37.63)	9.11±1.89 (6.74–11.35)	71±2 (69–73)	\	\	raised and projected	pointed*	Oberhummer et al. 2014

##### Ecology notes.

All tadpoles were collected from an artificial roadside drainage ditch (Fig. 5C) at night while feeding beneath the water surface. Upstream of the ditch is a narrow, slow-moving stream with many rocks covered by moss. The ditch was rocky with a sandy substrate. The maximum depth of this ditch was ~ 0.5 m. Branches of plants from the mountain side of the road extended over this ditch, however, sunlight did reach the water surface at certain times of the day. Tadpoles were observed in a still water stretch behind big rocks, or a small dam formed by submerged leaf litter. Three tadpoles at Stages 36–37 were collected on 30 May 2021 at 22:30 h, together with tadpoles of *Bo.shimentaina* and *Bo.nanlingensis* with an ambient air temperature of ~ 20 °C. Two tadpoles at Stages 2627 were collected on 16 November 2021 at 19:30 h with an ambient temperature of ~ 13 °C. Tadpoles were considered nocturnal because we did not encounter any tadpoles during the day. Male *Br.popei* frogs began their calling activities under rock crevices in this ditch in late July. Zhao et al. (2014) reported that the breeding season of *Br.popei* is July to September, and that their tadpoles (Stages 26–29) were collected in April and December. This indicates that the development of these tadpoles may be very prolonged, and it is likely that they can over winter. Interestingly, it is unknown why no tadpoles were collected during the breeding season both in this study and in Zhao et al. (2014).

#### ﻿ Boulenophrys shimentaina

Taxon classificationAnimaliaAnuraMegophryidae

6C1E7C4C-C6C2-5A74-BA16-069197FDE241

Fig. 2

##### Remark.

The following description is based on five tadpoles at Stages 25–28 (*N* = 5). Body ratio ranges represent all specimens. Raw measurements are given in Table 1.

##### Specimens examined.

CSUFT T10156 (Stage 25; Field voucher: MT06; GenBank accession number: ON209270) collected on 30 May 2021 from Tiantaishan (24.972277°N, 112.963394°E, ca. 1280 m a.s.l.), Mangshan, Hunan Province, China; and CSUFT T10277 (Stage 26, Field voucher: MT707; GenBank accession number ON209281), CSUFT T10279 (Stage 26; Field voucher: MT709; GenBank accession number: ON209264), CSUFT T10283 (Stage 28, Field voucher: MT713; GenBank accession number: ON209261); and CSUFT T10285 (Stage 27; Field voucher: MT715; GenBank accession number: ON209272) collected on 14 July 2021 from Xiangsikeng (24.937705°N, 112.990257°E, ca. 1530 m, a.s.l.), Mangshan, Hunan Province, China.

##### External morphology.

The body is oval and flattened above (BW/BL 51.3–55.0%, *N* = 5); the eyes are located dorsolaterally, and the pupils are round; the nares are oval, open laterally, closer to the eye than to the tip of the snout (NE/SN 62.5–71.4%, IND/IOD 67.6–71.9%, *N* = 5); the rims of nares are serrated, slightly raised from the body wall; the spiracle is sinistral, low on the left flank; the spiracle tube is short, free from the body at the tip and opens laterally (SS/BL 53.4–58.0%, *N* = 5); the anal tube opens medially, unattached to the ventral fin; the dorsal fin arises behind the body-tail junction while the ventral fin is connected to the trunk; the tail muscle is massive, taller than tail fins before reaching the 2/3 part of the tail length (TMH/MTH 50.0–55.6%, *N* = 5); the tail tip is bluntly pointed, the tail length accounts for 69.5–76.1% (*N* = 4) of the total length; the mouth is terminal and the oral disc is funnel-like (BW/ODW 65.2–77.2%, *N* = 5); four rows of oval submarginal papillae are visible on the upper lip, and five rows of oval submarginal papillae on the lower lip; keratodonts are absent; the upper jaw sheath is comb-like, exhibiting a small median notch; the lower jaw sheath is thin and sickle-shaped, weakly keratinized, and finely serrated.

##### Coloration.

The following description is based on a tadpole at Stage 27 (CSUFT T10285). In life, the background color of the body and tail is semi-transparent dark brown; the dorsum is pigmented pale brown which extends to the dorsal surface of anterior tail and gradually becomes golden; a distinct circled marking is present at the center of dorsum, forming a saddle with the background dark brownish coloration; the middle of the saddle is pigmented pale brown; and the neuromasts are distinctly visible. Laterally, the dorsal pattern extends to the region above the horizontal level of the spiracle on the trunk, and covers the whole lateral surface of head; the lateral surface of tail is pigmented brown; the tail and fins are covered with irregularly shaped pale golden spots, interspersed with dense dark brown speckles; the fins are semi-transparent; the anterior part of the dorsal fin is marbled with golden and dark brown speckles; the junction of the anterior half of the dorsal fin and the caudal muscle is pigmented dark brown, forming an incomplete line; the anterior part of the ventral fin and the anal tube exhibit minimal dark brown pigmentation; the posterior part of tail and fins are pigmented with dense dark brown markings. The ventral body is semi-translucent grey, pigmented with dark brown chromocytes, and is covered with dense small, indistinct milky-white speckles; the gills and gut coils are visible through the ventral skin; two large, milky-white spots are present on each side of the ventrolateral surface of head-body connection and are followed by a cluster of smaller spots. The oral disc is translucent milky white; the lateral and middle wings are covered with orangish pigmentation; the tips of the wings and the middle of the upper lip exhibit dark brown pigmentation; the submarginal papillae on lips are dark brown, and the narial rims are pigmented beige. The eye sclera is silver with black dots; the iris is orange sprinkled with black dots; and the spiracle is translucent without pigmentation.

**Figure 2.**
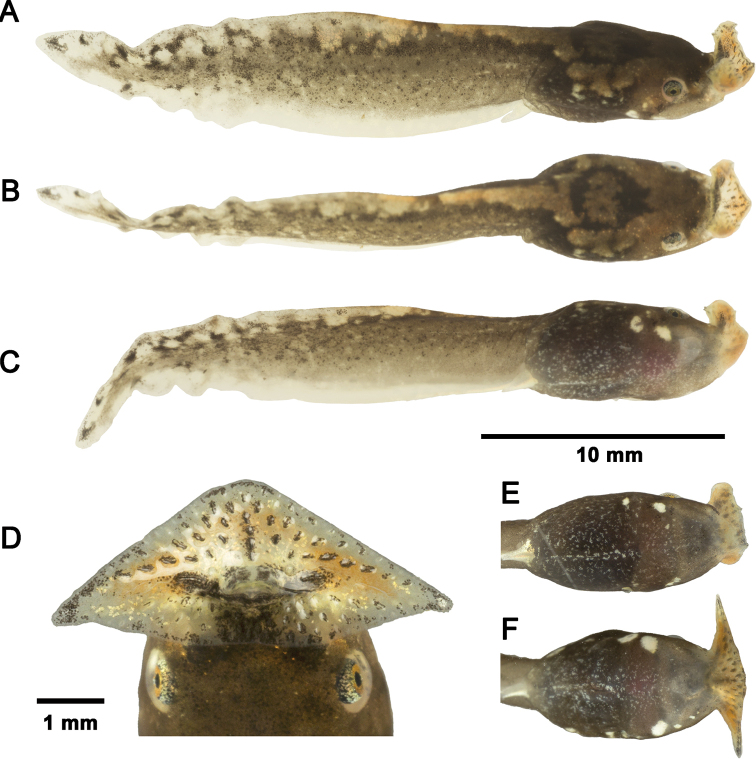
*Boulenophrysshimentaina* tadpoles **A–C** freshly dead tadpole CSUFT T10285 (Stage 27) lateral view, dorsal view, and ventral view **D** oral disc of tadpole CSUFT T10283 (Stage 28) in life **E** ventral pattern of tadpole CSUFT T10277 (Stage 26) in life; and **F** ventral pattern of CSUFT T10283 (Stage 28) in life. **E** and **F** share the same scale bar with **A–C**.

***Variation of coloration in life*.** The other four tadpole specimens match most of the descriptions above. However, the dorsum pattern of a saddle is not clearly visible in CSUFT T10156 and the dorsum is almost uniform pale brown in CSUFT T10177. The ventrolateral spots on head-body connection are very large in CSUFT T10283 (Stage 28, Fig. 2F), but smaller in CSUFT T10277 (Stage 26, Fig. 2E).

In preserved specimens, the pale brown pigmentation on the dorsal surfaces of the body and tail are still visible; the golden and orangish pigmentation fade to milky white; the white spots on each side of the ventrolateral surface of head-body connection become translucent; there is no orange pigmentation on the mouthparts, and prominent black pigmentation can be observed on the tail.

##### Comparisons.

The two distinct, conspicuous ventrolateral spots on ventrolateral surface of head-body connection could distinguish the tadpoles of *Bo.shimentaina* from most *Boulenophrys* tadpoles, including *Bo.fansipanensis*, which have a single spot visible on each side, and *Bo.rubrimera*, *Bo.hoanglienensis*, *Bo.jingdongensis*, *Bo.leishanensis*, *Bo.jiangi*, and *Bo.lushuiensis* with no ventrolateral spots; the ventral pattern of indistinct, small speckles on belly could distinguish *Bo.shimentaina* tadpoles from *Bo.lini*, which have dense large speckles (see Wang et al. 2014: fig. 5F). Furthermore, the tadpoles of *Bo.shimentaina* differs from *Bo.lushuiensis* by having a silver sclera with black dots (vs. black with golden pigments); and from *Bo.baishanzuensis* by having a pale brown pattern on dorsum (vs. uniformly brownish black).

Tadpoles of *Bo.shimentaina* could be distinguished from the syntopic *Boulenophrys* tadpoles in Mangshan (see below for the descriptions) by having a dark brown background coloration of body and tail (vs. pale brown in Bo.cf.ombrophila and *Bo.nanlingensis*), and a tail pattern of dense dark brown markings posteriorly (vs. several large brown spots along tail muscle in Bo.cf.ombrophila; and many brown speckles in *Bo.nanlingensis*). Further comparisons between *Bo.shimentaina* tadpoles and all megophryinid tadpoles identified based on molecular data are shown in Tables 2, 3.

##### Ecology notes.

A single tadpole at Stage 25 was collected on 30 May 2021, together with the tadpoles of *Bo.nanlingensis* and *Br.popei* from the road ditch (Fig. 5C) that was mentioned above in the *Br.popei* section. Four tadpoles at Stages 25–28 were collected together with tadpoles of *Bo.nanlingensis* and Bo.cf.ombrophila from a rocky, slow-flowing narrow stream (Fig. 5B) on 14 July 2021 at 23:20 h while nearby adult males were calling. As this stream is located near the mountain top, it is narrow and slow. There were low trees and bamboo on both sides of the stream, and many fallen logs lay across the stream with a rocky stream bed. This site was used by many species as a breeding site including *Bo.nanlingensis*, *Bo.shimentaina*, *Br.popei*, *Leptobrachellamangshanensis* (Hou, Zhang, Hu, Li, Shi, Chen, Mo & Wang, 2018), and *Quasipaaexilispinosa* (Liu & Hu, 1975). The tadpoles of *Bo.shimentaina* found in this stream were observed at night in an area with sandy substrate near the stream bank or in still water behind a small dam formed by submerged leaf litter. Sunlight could reach the surface of these areas at certain times during the day. While feeding beneath the water surface, the tadpoles rely on submerged leaf litter or rocks (Fig. 5A). Once disturbed, they hid quickly under the submerged leaf litter and emerged from the leaf litter after several seconds. In the still water area where these tadpoles were found, we also encountered many *Q.exilispinosa* tadpoles on the stream substrate, and a subadult newts, *Pachytritonxanthospilos* Wu, Wang & Hanken, 2012, hiding under submerged leaf litter. Male *Bo.shimentaina* frogs were observed calling from late June to August in Mangshan, and it was suggested that the breeding season of *Bo.shimentaina* is from April to August in Shimentai Nature Reserve, Guangdong Province (Lyu et al. 2020). It is not clear if tadpoles complete metamorphosis within a single year, and we didn’t collect any tadpoles of more advanced developmental stages.

#### ﻿ Boulenophrys cf. ombrophila

Taxon classificationAnimaliaAnuraMegophryidae

9015C633-CB00-5DD3-B201-12EDE0BE9963

Fig. 3

##### Remark.

The following description is based on four tadpoles at Stages 25–27 (*N* = 3) and Stage 36 (*N* = 1). Body ratio ranges represent all specimens except where specified. Raw measurements are given in Table 1.

##### Specimens examined.

CSUFT T10992 (Stage 25; field voucher: MT02; GenBank accession number: ON209283) collected on 3 June 2021; and CSUFT T10281 (Stage 26; field voucher: MT718; GenBank accession number: ON209275), CSUFT 10270 (Stage 36, field voucher: MT710; GenBank accession number: ON209267), and CSUFT T10272 (Stage 27, field voucher: MT712; GenBank accession number: ON209269) collected on 14 July 2021. All specimens were collected from Xiangsikeng (24.937705°N, 112.990257°E, ca. 1530 m, a.s.l.), Mangshan, Hunan Province, China.

##### External morphology.

The body is flattened and oval (BW/BL 52.9–54.8% at Stages 25–27, *N* = 3; and 51.0% at Stage 36, *N* = 1); the eyes are located dorsolaterally, the pupils are round; the nares are oval, opening laterally, closer to the eye than to the tip of the snout (NE/SN 60.0–73.3% at Stages 25–27, *N* = 3; and 82.4% at Stage 36, *N* = 1); the internarial distance is smaller than interorbital distance (IND/IOD 65.8–68.2% at Stages 25–27, *N* = 3; and 66.7% at Stage 36, *N* = 1); the rims of nares are serrated, slightly raised from the body wall; the spiracle is sinistral, low on the left flank, and opens posterolaterally; the spiracle tube protrudes posteriorly, free from the body at the tip (SS/BL 54.9–59.6% at Stages 25–27, *N* = 3; and 56.0% at Stage 36, *N* = 1); the anal tube opens medially, unattached to the ventral fin; the dorsal fin arises behind the body-tail junction while the ventral fin is connected to the trunk; the tail muscle is massive, taller than tail fins until reaching 2/3 of the tail length (TMH/MTH 45.8–53.3% at Stages 25–27, *N* = 3; and 48.3% at Stage 36, *N* = 1); the tail tip is usually sharply pointed (bluntly rounded in one small-sized specimen CSUFT T10992, Stage 25, TTL 20.9 mm); the tail length accounts for 72.4–75.6% of the total length at Stages 25–27 (*N* = 3), and 70.3% at Stage 36 (*N* = 1); the mouth is terminal and the oral disc is funnel-like (BW/ODW 61.5–69.2% at Stages 25–27, *N* = 3; and 61.4% at Stage 36, *N* = 1); three and four rows of short oval submarginal papillae are present on the upper and lower lips, respectively; keratodonts are absent; the upper jaw sheath is comb-like, exhibiting a small median notch, whereas the lower jaw sheath is thin and sickle-shaped, weakly keratinized, and finely serrated.

##### Coloration.

**Figure 3.**
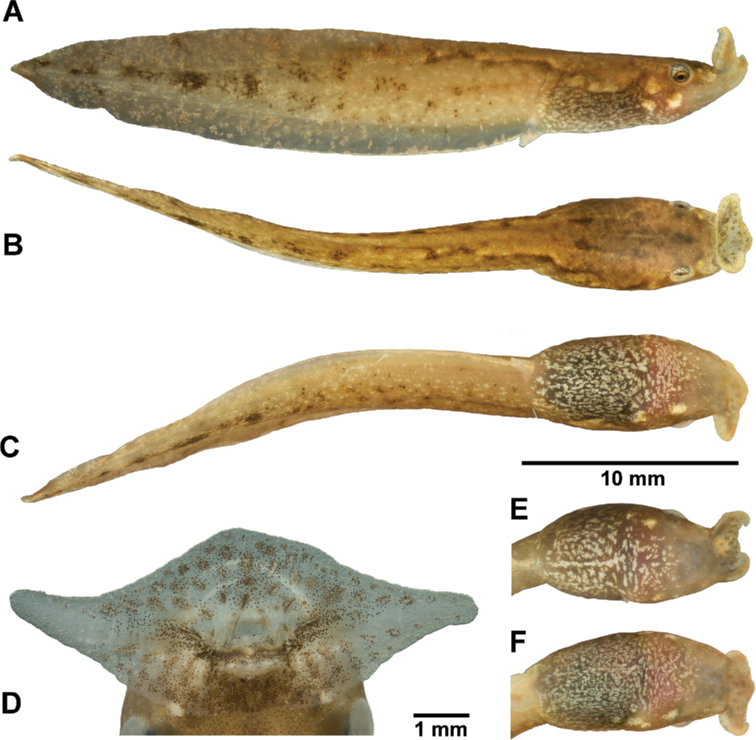
Boulenophryscf.ombrophila tadpoles **A–C** tadpole CSUFT T10272 (Stage 27) lateral view, dorsal view, and ventral view in life **D** oral disc of tadpole CSUFT T10270 (Stage 36) in preservative **E** ventral pattern of tadpole CSUFT T10281 (Stage 26) in life; and **F** ventral pattern of tadpole CSUFT T10270 (Stage 36) in life. **E** and **F** share the same scale bar with **A–C**.

The following description is based on a tadpole at Stage 27 (CSUFT T10272, Fig. 3A–C). In life, the background color of the body and tail is semi-transparent beige; the dorsal surface of the body is covered by a pale brown pattern that extends to the dorsal surface of the anterior part of the tail; a dark spot is present between the eyes and followed by a short beige vertical line on the anterior dorsum; the neuromasts are distinctly visible; and sparse dark brown markings alongside the vertical line and the dorsolateral neuromasts. Laterally, the dorsal pattern extends to above the horizontal level of the spiracle; three large, whitish, and golden pigmented spots are present behind the eyes on each side of the lower part of head-body connection, two of them are visible from the ventral view; the lateral surface of the tail and fins is covered by irregularly shaped pale golden spots, interspersed with whitish chromocytes which form short lines, and brown chromocytes which gather into large spots along the tail muscle at the posterior part of the tail; the fins are semi-transparent; the anterior part of the dorsal fin is marbled with golden and dark brown speckles; the anterior part of the ventral fin and the anal tube exhibit minimal brown pigmentation whereas the rest of fins that exhibits sparse dark brown speckles. The ventral body skin is translucent beige, covered by dense milky white speckles; the gills and gut coils are indistinctly visible through the ventral skin. The oral disc is translucent beige; the lateral and middle wings are covered by orange pigmentation; the submarginal papillae on lips are dark brown; the narial rims are yellow; the eye sclera is silver with black dots; the iris is bright orange sprinkled with black dots; the spiracle is translucent, with scattered golden pigmentation.

***Variation of coloration in life*.** Among the remaining three specimens examined, two tadpoles at Stages 26 and 36 match most of the coloration pattern of the description for CSUFT T10272 above (see Fig. 3E, F for ventral patterns). However, the dark spot between the eyes is not present in both; the vertical line is more distinct in CSUFT T10281, which extends from the middle of the eyes to the body-tail connection; and the vertical line in CSUFT T10270 is a bit longer than CSUFT T10272, which extend to the posterior dorsum. In the Stage 36 tadpole (CSUFT T10270), the hindlimbs are semi-transparent, and the legs are covered externally by brown chromocytes. A small-sized tadpole at Stage 25 (CSUFT T10992, TTL 20.9 mm) exhibited a significantly different coloration from other three tadpoles of larger body size (Stages 26–27, and 36; TTL 30.4–33.7 mm): the dorsal pattern is pale brown, with scattered dense dark melanocytes, especially at the vertebral line region; an inconspicuous orange V-shaped pattern between the anterior edges of the eyes and the median point of the upper lip; two orangish spots at the posterior edge of the eyes; the orangish pigmentation is also diffuse on the dorsal aspect of the body and tail; the ventral skin is almost clear, translucent milky, with sparse goldish speckles on the edge of the belly; the belly is covered with dense melanocytes, however, gut coils clearly visible through these melanocytes.

In preserved specimens, a fading of the dorsal pattern is observed; the golden spots on the lateral surfaces of the tail are not visible; the large spots on the anterior corner of the spiracle and gills become translucent, and the orange pigmentation on the lips disappears.

##### Comparisons.

The tadpoles of Bo.cf.ombrophila differ from the syntopic tadpoles of *Bo.shimentaina* by the semi-translucent beige background coloration of body and tail (vs. dark brown), a ventral pattern of dense milky white speckles on belly (vs. indistinct small speckles), and the pattern on tail of large spots along tail muscle (vs. dense markings posteriorly); from *Bo.nanlingensis* (see below for tadpole description) by the ventral pattern of dense whitish speckles (vs. sparse speckles), and the pattern on tail of several large spots (vs. many speckles).

Compared to other described *Boulenophrys* tadpoles where species identification is supported with molecular data, the tadpoles of Bo.cf.ombrophila differs by the ventral pattern of dense whitish speckles (vs. relatively sparse metallic blue speckles in *Bo.fansipanensis*; sparse whitish speckles in *Bo.jingdongensis*; sparse metallic grey blue speckles in *Bo.hoanglienensis*; and scattered with silver tiny patches in *Bo.lushuiensis*), and the tail pattern of several large spots along tail muscle (vs. few dark brown speckles in *Bo.fansipanensis*; absence of large spots in *Bo.jingdongensis*; many dark brown speckles in *Bo.hoanglienensis*; small black spots in *Bo.baishanzuensis*; few dark spots on posterior tail muscle in *Bo.jiangi*; small black spots on tail muscle in *Bo.leishanensis*; and dozens of small dark brown patches in *Bo.lushuiensis*). Further comparisons between Bo.cf.ombrophila tadpoles and all megophryinid tadpoles that were identified based on molecular data are shown in Tables 2, 3.

##### Ecology notes.

One observed breeding site of Bo.cf.ombrophila was a relatively broad wetland crossed by a small shallow creek. Several water sources from the gentle slope of the bamboo forest fed this creek and made the entire area very wet. This breeding site was muddy, covered with leaf litter and fallen logs. The creek was narrow and slow flowing with maximum depth of 0.2 m. Some fallen logs blocked the creek and created still water areas. Only male Bo.cf.ombrophila and *Q.exilispinosa* were observed calling in this site during our visits from May to August, and in November. The potential predator of these frogs, an aquatic snake *Opisthotropischeni* Zhao, 1999 which was observed once, in July, in this creek. A single small-sized tadpole specimen (CSUFT T10992, TTL 20.9 mm) at Stage 25 was collected from this site while the male frogs were heard calling before a heavy rainstorm on 3 June 2021 at 19:30 h at dusk. Three tadpoles were collected from the rocky area (Fig. 5B, mentioned above in the *Bo.shimentaina* section) 20 m downstream of this creek together with tadpoles of *Bo.shimentaina* and *Bo.nanlingensis*. Interestingly, male Bo.cf.ombrophila frogs were not observed calling in the rocky area, and the other two species did not breed in this wetland. This indicates a different microhabitat preference between these congeneric species. The breeding season of Bo.cf.ombrophila ends in mid-July in Mangshan. It is not clear if tadpoles will complete metamorphosis during the year.

#### ﻿ Boulenophrys nanlingensis

Taxon classificationAnimaliaAnuraMegophryidae

28838EA6-578E-5E4C-8A3D-908728F54FF8

Fig. 4

##### Remark.

The following description is based on 14 tadpoles at Stages 25–29 (*N* = 12), and 34–35 (*N* = 2). Body ratio ranges represent all specimens except where specified. Raw measurements are given in Table 1.

##### Specimens examined.

CSUFT T10144 (Stage 25; field voucher: MT04; GenBank accession number: ON209279) collected on 30 May 2021; and CSUFT T10302 (Stage 25, field voucher: MT722; GenBank accession number: ON209280), and CSUFT T10303 (Stage 25, field voucher MT723; GenBank accession number: ON209277) collected on 19 July 2021 from Tiantaishan (24.972277°N, 112.963394°E, ca. 1280 m a.s.l.); CSUFT T10261 (Stage 25; field voucher: MT701; GenBank accession number: ON209263), CSUFT T10262 (Stage 25; field voucher: MT702; GenBank accession number: ON209268), CSUFT T10273 (Stage 28, field voucher: MT703, GenBank accession number: ON209278), CSUFT T10991 (Stage 27; field voucher: MT711; GenBank accession number: ON209265), and CSUFT T10284 (Stage 25, field voucher: MT714; GenBank accession number: ON209271) collected on 14 July 2021; and CSUFT T10986 (Stage 35, field voucher: MT1106; GenBank accession number: ON209285) and CSUFT T10969 (Stage 34, field voucher: MT1109; GenBank accession number: ON209274) collected on 19 November, 2021 from Xiangsikeng (24.937705°N, 112.990257°E, ca. 1530 m, a.s.l.); and CSUFT T10376 (Stage 27, field voucher: MT756; GenBank accession number: ON209273), CSUFT T10377 (Stage 27, field voucher: MT757; GenBank accession number: ON209262), CSUFT T10378 (Stage 28, field voucher: MT758; GenBank accession number: ON209282), and CSUFT T10379 (Stage 29, field voucher: MT769; GenBank accession number: ON209266) collected on 28 July 2021 from Guizizhai (24.952750°N, 112.960470°E, ca. 1210 m a.s.l.), Mangshan, Hunan Province, China.

##### External morphology.

The body is elongated, oval, and flattened above (BW/BL 51.2–60.4% at Stages 25–29, *N* = 11; and 52.8–54.5% at Stages 34–35, *N* = 2); the eyes are located dorsolaterally, and the pupils are round; the nares are oval, closer to the eye than to the tip of the snout (NE/SN 55.6–80.0% at Stages 25–29, *N* = 12; and 57.9–62.5% at Stages 34–35, *N* = 2); the internarial distance is smaller than interorbital distance (IND/IOD 61.5–71.0% at Stages 25–29, *N* = 12; and 68.1–69.2% at Stages 34–35, *N* = 2); the nares open laterally; the rims of nares are serrated, slightly raised from the body wall; the spiracle is sinistral, low on the left flank, and opens posteriorly; the spiracle tube is short and slightly protrudes posteriorly (SS/BL 54.8–62.7% at Stages 25–29, *N* = 12; and 59.3–63.6% at Stages 34–35, *N* = 2). The anal tube opens medially and is unattached to the ventral fin; the dorsal fin arises behind the body-tail junction, and the ventral fin is connected to the trunk. The tail muscle is massive, deeper than tail fins before reaching the maximum tail height (TMH/MTH 43.4–63.0% at Stages 25–29, *N* = 11; and 50.7–54.5% at Stages 34–35, *N* = 2); the tail tip is pointed, the tail length accounts for 69.5–76.1% (at Stages 25–29, *N* = 11) and 73.1–74.4% (at Stages 34–35, *N* = 2) of the total length; the mouth is terminal and the oral disc is funnel-like (BW/ODW 63.3–79.6% at Stages 25–29, *N* = 12; and 73.1–85.7% at Stages 34–35, *N* = 2); four and five rows of short oval submarginal papillae can be observed on the upper and lower lips, respectively; keratodonts are absent; the upper jaw sheath is comb-like, exhibiting a weak median notch; the lower jaw sheath is thin and sickle-shaped, weakly keratinized, and finely serrated.

##### Coloration.

**Figure 4.**
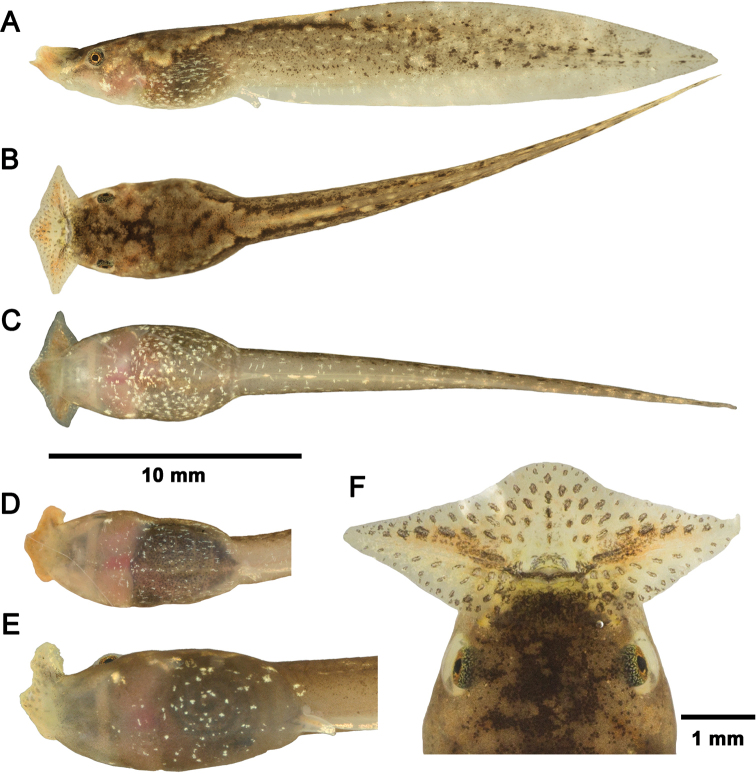
*Boulenophrysnanlingensis* tadpoles in life **A–C** tadpole CSUFT T10303 (Stage 25) lateral view, dorsal view, and ventral view **D** ventral pattern of tadpole CSUFT T10262 (Stage 25) **E** ventral pattern of tadpole CSUFT T10273 (Stage 28); and **F** oral disc of tadpole CSUFT T10261 (Stage 25). **D** and **E** share the same scale bar with **A–C**.

The following description is based on a tadpole at Stage 25 (CSUFT T10303, Fig. 4A–C). In life, the background color of the body and tail are semi-transparent grey; the dorsal surface of the body is covered by a pale brown pattern that extends to the dorsal surface of the anterior part of the tail; roughly symmetrical dark brown pigmentation can be observed on the dorsal body; and the neuromasts are distinctly visible. Laterally, the dorsal pattern extends to above the horizontal level of the spiracle; the lateral surface of the tail is pigmented brown, interspersed with pale golden spots and irregular dark brown speckles; the fins are semi-transparent and scattered with pale golden spots; the anterior part of the dorsal fin is marbled with golden and dark brown speckles, with the dark brown speckles forming an incomplete line; the anterior part of the ventral fin and the anal tube, lacks brown pigmentation but with sparse golden speckles. The ventral surface of the body is semi-transparent grey; the gills appear pink through the ventral skin; two large gold-pigmented white spots are present at ventrolateral head-body connection; the gut coils are distinctly visible through the ventral skin, the belly is scattered with small whitish speckles; the oral disc is translucent beige; the lateral and middle wings are covered by orangish pigmentation; the submarginal papillae on lips are dark brown; the narial rims are beige; the eye sclera is silver with black dots; the iris is copper-colored sprinkled with black dots, comparable to the iris coloration in adults; and the spiracle is translucent.

**Figure 5.**
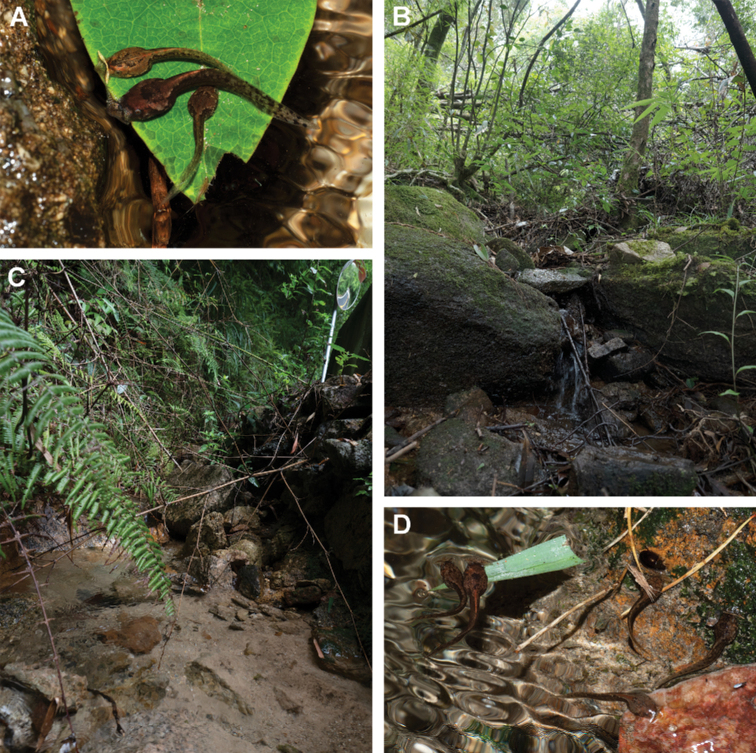
Megophryinid tadpoles and their habitats in Mangshan, Hunan Province, China (**A**) Non-collected *Bo.shimentaina* tadpole (middle) feeding together with two unrecognized tadpoles in its habitat (**B**); and tadpole habitat (**C**) of a mixed-species assemblage beside a forest road, with unrecognized tadpoles feeding together (**D**).

***Variation of coloration in life*.** The dorsal pattern coloration in tadpoles of *Bo.nanlingensis* is subject to significant variation both in same stages and between stages. At Stage 25, a small-sized tadpole (CSUFT T10144, TTL 18.7 mm) exhibits a yellowish dorsum with pale orange blotches, and dark brown pigmentation present posteriorly; another small tadpole (CSUFT T10284, TTL 18.9 mm) displays a brown dorsum with whitish patterns on the dorsolateral surfaces of the trunk and these extend to the tip of the tail. The coloration of the medium-sized tadpoles at Stage 25 (CSUFT T10261, TTL 25.1 mm; and CSUFT T10262, TTL 27.2 mm) and a broken-tailed individual at Stage 25 (CSUFT T10302) correspond to the dorsal pattern of CSUFT T10303 described above. However, the shape and coverage of dark brown markings varies between individuals. At later Stages 27–29, three medium-sized tadpoles (CSUFT 10377, Stage 27, TTL 28.1 mm; CSUFT T10376, Stage 27, TTL 24.8 mm; and CSUFT 10378, Stage 28, TTL 26.9 mm) exhibit a bi-colored dorsum, which is anteriorly pale brown and posteriorly inconspicuous dark brown. Tadpoles with relatively larger size at both early Stages 27–28 and advanced Stages 34–35 (CSUFT T10273, Stage 28, TTL 35.7 mm; CSUFT T10991, Stage 27, TTL 39.1 mm; CSUFT T10986, Stage 35, TTL 40.1 mm; and CSUFT T10969, Stage 34, TTL 34.4 mm) exhibit a uniform brownish dorsum coloration with almost invisible markings. Two tadpoles, CSUFT T10144 (Stage 25, TTL 18.7 mm) and CSUFT T10379 (Stage 29, TTL 27.8 mm) exhibit pale yellowish dorsum with orange pigmentation, which are indistinguishable from the small-sized Bo.cf.ombrophila tadpole (CSUFT T10992, TTL 20.9 mm). A tadpole at Stage 27 (CSUFT T10376, TTL 24.8 mm) with a mid-vertical line on dorsum is similar with that of larger Bo.cf.ombrophila tadpoles (Stages 26–27, and 36; TTL 30.4–33.7 mm). However, they were not collected form the same site as Bo.cf.ombrophila tadpoles. A large-sized tadpole at Stage 35 (CSUFT 10986, TTL 40.1 mm) showed a ventral pattern of large spots on belly that was different with other specimens. For tadpoles at Stages 34–35 (CSUFT T10969, and CSUFT T10986), the hindlimbs are semi-transparent, the outer aspect of the legs is pigmented yellow and interspersed with brown chromocytes on top.

In preserved specimens, a fading of the dorsal pattern is observed; the tail is translucent with sparse dark-brown pigmentation; the orange pigmentation on lips is no longer visible; the whitish speckle on the ventral surface and the nares are translucent.

##### Comparisons.

The variation of dorsum pattern makes the tadpoles of *Bo.nanlingensis* are sometimes confused with the syntopic tadpoles of Bo.cf.ombrophila. Usually, the ventral pattern of sparse speckles (vs. dense speckles) and the tail pattern of many small speckles (vs. large spots) could distinguish them. An exception is the small-sized tadpole CSUFT 10144 (Stage 25, TTL 18.7 mm), which bears almost the same pattern as a small-sized Bo.cf.ombrophila tadpole CSUFT T10992 (Stage 25, TTL 20.9 mm). The tadpoles of *Bo.nanlingensis* differ from the syntopic *Bo.shimentaina* tadpoles by the pale brownish background coloration of the body and tail (vs. dark brown), the ventral pattern of sparse speckles (vs. dense small speckles), and the tail pattern of small dots (vs. large speckles).

Compared to other described *Boulenophrys* tadpoles where species identification is supported by molecular data, the tadpoles of *Bo.nanlingensis* differs by the presence of ventrolateral spots on each side of head-body connection (vs. absent in *Bo.jingdongensis*, *Bo.hoanglienensis*, *Bo.leishanensis*, and *Bo.lushuiensis*); the tail pattern of many brown speckles (vs. small spots on tail muscle in *Bo.leishanensis*; few dark spots on posterior tail muscle in *Bo.jiangi*; and small white and black dots in *Bo.baishanzuensis*). Further comparisons between *Bo.nanlingensis* tadpoles and all megophryinid tadpoles that were identified based on molecular data are shown in Tables 2, 3.

##### Ecology notes.

Tadpoles of *Bo.nanlingensis* were discovered in all collection sites during our field surveys in 2021, which perhaps implies that this species has larger population size, or it might exhibit less microhabitat specificity. Besides the three sites mentioned above, four *Bo.nanlingensis* tadpoles were collected from a relatively wide stream (3–5 m wide), with a maximum depth of 0.5 m. An adult male was observed calling under rocks near the stream bank with its feet standing in shallow water on 28 July 2021.

The male calling activities of *Bo.nanlingensis*, which began in late July, had increased during our visit in November in Mangshan. It seems the newborn larva would have to over-winter. Thus, we suspected the tadpoles of early Stages 25–29 collected in May and July were born in the previous year. Two tadpoles at advanced Stages 34–35 were collected on the 19^th^ of November. Considering tadpoles in late stages develop relatively fast (Grosjean, 2003; TQ, personal observation). It was likely these advanced tadpoles would finish metamorphosis in cold season at the beginning of the next year. However, this assumption needs further confirmation because the cold weather and scarce food in winter may not be suitable for the survival of froglets.

## ﻿Discussion

In this study, we attempted to identify sympatric megophryinid tadpoles using external morphology and color patterns, especially ventral patterns. However, our sample size was small. Tadpoles of *Bo.shimentaina* and Bo.cf.ombrophila bearing stable and distinct ventral pattern were collected from a single site. It is not clear if the color pattern may differ between sites. In *Bo.nanlingensis*, the color pattern varied between different body size ranges rather than stages or collection sites. This provides new insight into that the coloration pattern perhaps should be classified by body size ranges in megophyinid tadpoles, and not only development stages. Diagnostic larval characters for delineating megophryid genera are still insufficient except for the ventrolateral pattern in *Brachytarsophrys*. However, there are characters shared within genera, such as the rim of nares is “tubular” in *Ophryophryneelfina* (Poyarkov et al. 2017), and *O.microstoma* Boulenger, 1903 (tadpoles described by Grosjean, 2003 without molecular data), but this rim is “serrated” in *Boulenophrys* tadpoles, described both in this study and in Tapley et al. (2020b); or the oral disc is “hastate shaped” in *Atympanophrysgigantica* (Tapley et al. 2020b), which has not been reported in other genera.

We failed to find any *Xenophrysmangshanensis* adult or larva during our field surveys, despite Mangshan being its type locality. However, it was reported to be in sympatry with *Bo.nanlingensis* and *Br.popei* in Guangdong Province (Wang et al. 2019). As earlier mentioned under the ecology notes for Boulenophryscf.ombrophila, sometimes larvae maybe washed downstream where adult frogs are not thought to be present. Therefore, if megophryinid species occur in sympatry with others, the tadpole identification without molecular data should be re-considered. Perhaps, the tadpole specimens previously described as *X.mangshanensis*, for example, by Fei and Ye (2016) should be re-examined after molecular identification.

The presence of *Bo.kuatunensis* and *Bo.brachykolos* in Mangshan was not confirmed in this study, which agrees with the conjecture proposed by Liu et al. (2018) and Tapley et al. (2017) that both species are restricted to their type localities. However, taxonomic revisions need adequate field surveys and detailed examination of museum specimens (Qi et al. 2021). This study provides an example of using tadpole identification to document the presence of species where adults may not always be visible. This proved particularly useful here as the tadpoles of megophryinid frogs at this site appear to be relatively slow to develop, and they could always be found outside the breeding season.

## Supplementary Material

XML Treatment for Brachytarsophrys popei

XML Treatment for Boulenophrys shimentaina

XML Treatment for Boulenophrys cf. ombrophila

XML Treatment for Boulenophrys nanlingensis
